# Researching authoritarian smart cities from below

**DOI:** 10.1177/29768640251375573

**Published:** 2025-09-12

**Authors:** Jasmin Dall’Agnola

**Affiliations:** Institut für Kommunikationswissenschaft und Medienforschung (IKMZ), 27217 Universität Zürich

**Keywords:** Authoritarianism, smart cities, fieldwork, CCTV cameras, Central Asia

## Abstract

Authoritarian smart cities are often studied from afar, through policy papers, media discourse, or elite perspectives. While important, such approaches tend to reproduce state-centric narratives, offering little insight into how residents actually experience these cities. In this commentary, I argue that to truly understand authoritarian smart cities, we must go beyond ‘repoliticising’ them and start ‘rehumanising’ them. Rehumanising demands more than critique from a distance; it requires grounded, adaptive, and ethically attuned fieldwork that engages directly with how people navigate and make sense of smart city technologies in their daily lives. Yet gaining such access in authoritarian contexts is far from straightforward. For that reason, and drawing on my ethnographic fieldwork in Kazakhstan, Kyrgyzstan, Tajikistan, and Uzbekistan in 2022, in this commentary, I offer three practical strategies for studying authoritarian smart cities from below: (1) strategic questionnaire; (2) relational ethics; and (3) tactical flexibility.

## Introduction

In June 2023, Kyrgyzstan's President Sadyr Zhaparov (2023) stood before an expectant crowd near Lake Issyk to unveil his vision for Asman City, the country's first greenfield smart city: ‘Asman will become the economic engine of our nation’, he proclaimed. Yet just one year later, construction has stalled, financing remains murky, and public scepticism is growing (VOA News, 2024). Like other top-down smart city initiatives in authoritarian contexts, such as NEOM in Saudi Arabia (Brown and Jones, 2025), Asman now stands not as a beacon of innovation, but as a symbol of the disconnect between techno-utopian rhetoric and lived urban realities.

Framed as solutions for efficiency, sustainability, and modernisation, authoritarian smart cities often function as tools of legitimation, centralisation, and control (Akbari, 2022; Da Vinha, 2024). Yet despite their proliferation, they remain under-researched and under-theorised. What we know tends to rely on state-authored documents, sanitised media reports, or outsider commentary based on dissidents’ accounts and speculative critique (Vila Seoane and Velasco, 2024). While valuable, these sources often reproduce state-centric narratives or critiques from afar, offering limited insight into the everyday experiences of authoritarian smart cities.

To address this gap, I argue that we need to move beyond ‘repoliticising’ the authoritarian smart city (Akbari, 2022) to ‘rehumanising’ it. While this notion has been explored in democratic contexts in relation to citizenship and social justice (Kitchin, 2019), in authoritarian smart cities, rehumanising means foregrounding the everyday lives, struggles, and agencies of those who inhabit these cities, rather than focusing solely on the visions of those who design or impose them. This requires ethically grounded fieldwork that engages directly with residents’ lived experiences, an undertaking that is far from straightforward in authoritarian settings. For that reason, and drawing on my ethnographic fieldwork in Kazakhstan, Kyrgyzstan, Uzbekistan, and Tajikistan in 2022, in this commentary, I offer three practical tips for studying authoritarian smart cities from below: (1) strategic questionnaire; (2) relational ethics; and (3) tactical flexibility.

## Strategic questionnaire

When researching authoritarian smart cities, it is essential to craft questions that invite open dialogue rather than those that might inadvertently lead research participants to incriminate themselves. This is particularly important in Central Asia, where the region's five countries, Kazakhstan, Kyrgyzstan, Tajikistan, Turkmenistan, and Uzbekistan, have transitioned from initial post-Soviet democratic experiments to varying degrees of authoritarian rule (Baer and Dall’Agnola, 2025). Even seemingly innocent questions can expose both participants and researchers to follow-up questioning by security services, intimidation, detention, or worse – risks that are particularly severe for locals who cannot leave the country (Dall’Agnola, 2023; Norov, 2024).

To avoid these risks, I reached out to the Central Asia Barometer (CAB), a local polling centre based in Kyrgyzstan's capital, Bishkek, to pre-test some of the more sensitive questions ahead of my fieldwork. With years of experience navigating political sensitivities, CAB routinely adapts surveys to minimise the above-mentioned risks for respondents and researchers (Ysmanova, 2024).

In preliminary consultations with their partner institutions across the region, local partners flagged the question I had designed on public attitudes towards CCTV cameras. Their advice: reframe and focus it solely on the camera's role in crime prevention to avoid political controversy. This experience of reviewing and refining my camera question with CAB's local partners proved essential in shaping how I later designed the questionnaire for my interviews and focus groups in Almaty, Bishkek, Dushanbe, and Tashkent. I deliberately avoided terminology or tones that might suggest smart city technologies were being used by local authorities to monitor or suppress civil society. Instead, I designed open-ended questions around interviewees’ experiences with smart city technologies they interacted with daily, such as smartphones, e-wallets, delivery services, and video cameras.

Moreover, I placed the camera question ‘How do you feel about CCTV cameras in public spaces?’ towards the end of the questionnaire and introduced it with a brief story emphasising the cameras’ roles in traffic regulation and monitoring of reckless drivers. While this framing portrayed cameras in a more positive light, it did not prevent some participants from voicing critical views on their use (Dall’Agnola, 2025). Participants seemed more at ease discussing smart city technologies and broader issues related to authoritarian governance when they were not directly prompted to express political views concerning their government.

## Relational ethics

Just as questionnaires must be strategically designed to fit authoritarian contexts, so too must our ethical frameworks. Fieldwork in authoritarian smart cities requires a relational approach to ethics, one that is attentive to local power dynamics and the real risks participants face.^1^

Protecting participants often means bending or abandoning formal protocols that might endanger or retraumatise them. For example, standard Western ethics procedures, such as signing formal consent forms, can alienate potential respondents in the authoritarian countries of Central Asia, where such acts often evoke memories of secret police and state control (Dall’Agnola, 2023; Gafu and Parameter, 2025). Instead, I adopted a more culturally and politically attuned approach: recording only anonymised verbal consent when participants felt comfortable, and allowing people to speak entirely off the record when needed. In most cases, the simple fact that someone chose to show up and engage in a conversation with me was itself a meaningful form of consent.

This flexible, trust-based approach shaped not only how I obtained consent, but how I interpreted responses. During one interview in Almaty, a security guard explained his feelings about CCTV cameras. ‘Sometimes they [the cameras] work, sometimes they don’t’, he said. ‘When something happens to me, they don’t work. But when they need them’, he added, offering a dry smile and pointing to the sky to indicate the authorities, ‘then the cameras work’. His subtle, symbolic gesture said far more than direct political criticism could have. It was an indirect, but unmistakable expression of mistrust towards his authorities’ use of smart city technologies.

Such moments highlight that building trust means giving respondents the choice to decide whether and how they want to express their concerns. Unlike the performative ‘vox pop’ interviews often seen on TV, where strangers are asked by journalists to criticise authoritarian leaders on camera, ethnographic work must allow for ambiguity, silence, and indirect forms of resistance. Relational ethics in this context means more than avoiding harm. It requires knowing when not to ask, when to soften, and when to let a gesture speak for itself.

Relational ethics also extends to post-fieldwork writing. Many scholars studying authoritarian smart cities resort to self-censorship to safeguard themselves, their families, and their participants from potential retaliation. This includes limiting their online presence, avoiding being photographed at high-profile events, withholding controversial findings, or even publishing under pseudonyms (Norov, 2024). Such self-censorship is a necessary, if painful, compromise, one I experienced firsthand. For the first two years after my fieldwork, I deliberately postponed publishing findings on the authoritarian nature of smart city technologies; instead, I focused on less risky topics such as fieldwork methods (Dall’Agnola, 2023). Consequently, early-career researchers with firsthand experience are often unable to fully claim their expertise, while more established scholars, who rarely visit the region, remain the dominant, untouchable ‘experts’.

## Tactical flexibility

Just as ethical frameworks must be adapted to local realities in authoritarian smart cities, so too must research logistics. Instead of relying on formal field access or institutional affiliations, I drew on informal networks of trust and local collaborators to guide my movements through Almaty, Bishkek, Dushanbe, and Tashkent. When participants worried about being seen with me in public, I held interviews in more discreet, private settings, such as their homes, a trusted friend's house, or rented private rooms in community centres. Sometimes, I reframed interactions entirely, not as formal interviews, but informal conversations about daily experiences with technology. This often meant abandoning my planned structure. Questions were skipped or reworded on the spot; audio recording was replaced by hand-written notes; timelines stretched to match participants’ comfort levels. Tactical flexibility also extends to language: while I conducted interviews in Russian, which I speak fluently, some participants preferred to communicate in local languages unfamiliar to me. In those cases, their multilingual colleagues often provided on-the-spot translations.

Building on this adaptability, I also adopted a readiness-to-interview mindset at all times. I carried my materials with me wherever I went, prepared to engage whenever trust emerged organically. Some of the most insightful conversations happened in moments I had not planned for. One such exchange took place on a flight from Almaty to Astana, when a casual chat with my seatmate, an IT engineer in his mid-20s, evolved into a spontaneous interview. Asked about CCTV cameras, he said bluntly: ‘They don’t prevent accidents, they function more like a punitive tool. 80% of these cameras are just there to punish us’. It was a clear-eyed critique of how smart city technologies operate not as protective mechanisms, but as instruments of state control. That conversation, held above the clouds amid the hum of engine noise, reminded me that in authoritarian smart cities, trust may surface in the most unexpected places, and it is the researcher's job to be ready when it does.

## Conclusion

Authoritarian smart cities are often studied from afar, through policy papers, media discourse, or the perspectives of political elites. But if I have learned anything from my fieldwork in Central Asia in 2022, it is that to understand how smart city technologies shape everyday life, we need to study them from below: attuned to how people living within these systems navigate, resist, or quietly repurpose the smart infrastructures around them.

For me, this meant embracing strategic questionnaires, relational ethics, and tactical flexibility, not as abstract principles, but as essential tools for capturing everyday experiences in authoritarian smart cities. These strategies helped me listen more carefully, adapt more responsively, and surface stories that official accounts often silence. They enabled me to reveal how people negotiate, subvert, and make sense of the authoritarian smart city on their own terms.

In that sense, rehumanising the authoritarian smart city has meant learning to see beyond its spectacle and control, and to recognise the subtle, everyday forms of agency people still enact. I have encountered them in words, in gestures, in silence, and sometimes 30,000 feet above the ground.

## Notes

1. Relational ethics, as I use the term here, is care-based rather than rule-based (Hakkim, 2022). It prioritises trust, empathy, and situational judgement over rigid adherence to Western research protocols.
